# Progress in Psychiatry

**Published:** 1903-07-25

**Authors:** 


### Progress in Psychiatry.

**Amaurotic Family Idiocy.**—Professor Bernard Sachs, of New York, gives an account[1] of amaurotic family idiocy, which is an expansion of the views propounded by him previously in 1887, in an article on "Arrested Cerebral Development." The clinical description then given has been endorsed by Hirsch, Peterson, and others. Bulbar disturbances with difficulty of deglutition have been met with as a grave symptom in cases observed in recent years. The case recorded by Sachs is that of a boy aged two years and seven months, born of Hebrew parents, and the third child of the family. The eldest is healthy and aged six years. The second child died at the age of 16 months. It was blind for some months preceding its death. It could never sit up straight, never uttered a sound, had no spasms, but was feverish at times. It was probably afflicted with amaurotic family idiocy. The third child is the present case. At birth and for some time afterwards the child appeared to be normal. It vomited, however, a great deal, and as time passed on it was found to be incapable of sitting upright, while it was not bright like other children. When about eight months old its sight began to fail, and at the age of one year it was practically blind (amaurosis). Typical degenerative changes in the macula lutea of the eye were made out. The child grew generally weak, contractures developed in the muscles of the upper and lower limbs, and difficulty of swallowing was manifested. The child died in an emaciated condition, its bodily temperature during the last two weeks of life being, as a rule, subnormal. The diagnosis of amaurotic family idiocy could be made with certainty in the present case. The autopsy showed a small brain weighing 784 grammes, the cerebral cortex was so firm "that the knife grated as it passed through it," the convolutions were narrow, and there were fissural peculiarities which indicated a low type of brain. The fluid in the pia-arachnoid meshes was markedly increased. The pituitary body was not enlarged. Serial sections of the spinal cord showed some degeneration of the crossed and direct pyramidal parts. Much more striking was the change in the grey matter of the central nervous system. The changes were found to be the same in the cerebral cortex, in the cranial nerve nuclei, and in the grey matter of the cord from the cervical to the lumbo-sacral region. These degenerative changes, as shown by Nissl's method, affected chiefly the larger ganglion cells throughout the entire extent of the central nervous axis. "In the cortex as well as in the anterior cornua of the spinal cord there is not a normal ganglion cell to be seen." The cell-body was altered, the cell-protoplasm had become disintegrated, leaving a more or less homogeneous mass, the nucleus had been shifted to the periphery and extruded altogether from the cell. The disintegration of the cell body was complete in many specimens, and the pericellular spaces were enlarged. Similar microscopic changes were found in a recent case by Hirsch.[2] The inference to be drawn from microscopic investigation is that the morbid process in amaurotic family idiocy affects largely and primarily the entire grey matter of the brain (cerebral hemispheres) and spinal cord, the degeneration of the pyramidal tracts being secondary. Sachs concludes that some initial or inherited defect of vitality of the nervous system, combined with the action of an unknown toxic agent, constitutes the two-fold ætiological cause of amaurotic family idiocy. There is no treatment for this disease, the termination being fatal.

**The Mental State of the Consumptive.**—That abnormal mental states are produced by chronic toxæmic conditions, such as alcoholism, is generally acknowledged. Chronic dyspeptic conditions often give rise to "auto-intoxication" from the bowel, a process which brings about headache, irritability, and even melancholic depression of spirits. Many observers have noted a peculiar mental "diathesis" associated with the taint of consumption. Dr. Felix Regnault[3] states that the most salient psychological manifestations of phthisis are hyperexcitability, optimism, egoism, and sentimentality. Though afflicted with a serious disease, the patient feels no pain in the early and intermediate stages, but, on the contrary, he feels buoyant. Even when made aware of his condition he is not downcast or demoralised, because his feelings of organic well-being deceive him. Moreover, his appetite is excellent, and if, under medical advice, he betakes himself to a sanatorium, the absence of work, worry, and anxiety during his sojourn adds to his insouciance. Sexual hyperexcitability is characteristic of many patients; these are generally subfebrile cases. Tuberculosis can add its psychological impress to other diseases—*e.g.*, in cases of myxœdema with apathy and mental dulness the development of tuberculosis will render the patient lively and excitable in character though it will not make him more intelligent. Professor Jules Voisin[4] stated that a psychical difference existed between patients with pulmonary tuberculosis and those with gastro-intestinal tuberculosis; he could distinguish the two classes of patients in his wards at the Salpêtrière, for the former were usually euphoric (possessed of a feeling of well-being) but the latter were rarely so. M. Berillon pointed out[5] that in the prodromal and early stages of their affection consumptive patients lacked strength of will, were easily led, and their minds tended to vacillate from project to project with great facility but realisation seldom followed. Later, when the disease was well established and pyrexia was present, a peculiar form of egoism was liable to develop. This egoism has in some instances made them leave wills to their successors designed in such a manner as to flatter the vanity of the testator. M. Lépinay,[6] a veterinary surgeon, said that before the appearance of the clinical symptoms of tuberculosis there occurred psychological symptoms of a special kind, which, though not pathognomonic, might yet lead to suspicion of impending tuberculosis. This had been observed, especially in cows, in the form of nervous disturbances affecting both the digestive and the sexual functions. The animal ate dirt and bits of wood, and displayed abnormal sexual excitement like one in heat. Such animals when killed showed signs of incipient tuberculosis. Pups affected with tuberculosis lost their affection and docility and became snappish and irritable, while abnormal sexual excitement was also manifested.

**Arterio-sclerosis and Mental Disease.**—Adolph Meyer,[7] while accepting the theory of a pathological connection between mental (cerebral) disease on the one hand and arterio-sclerosis with renal disease on the other, thinks the frequency of the association has been exaggerated. Arterio-sclerosis of the heart and aorta was exceedingly frequent in the insane, but it was not often that mental disorders could be directly ascribed to such lesions. This could only be done in case of arterio-sclerosis of the brain itself, and even here many difficulties were encountered. Arterio-sclerosis of the brain was in some persons associated with loss of memory of the immediate past, and transitory mental confusion or delirium. Sometimes such persons were liable to give way to acts of petty larceny, of arson, or of sexual misconduct. Mental disease occurring at that period of life when arterio-sclerosis was most common showed no difference in its nature and course that could not be fairly accounted for by the "diminished vital resistance" of later life. In short, there was little justification for speaking of an arterio-sclerotic insanity, though it was possible to recognise clinically both senile and presenile mental involution associated with arterio-sclerosis. Naturally enough, adds Dr. Meyer, there is no specific treatment for cases in which the underlying condition is arterio-sclerosis, but the knowledge of the existence of arterio-sclerosis is chiefly of value in prognosis.

[1] Jour. of Nervous and Mental Disease, Jan., 1903. [2] Ibid., 1898. [3] Archives de Neurologie, Jan., 1903. [4] Ibid. [5] Ibid. [6] Ibid. [7] New York Med. Rec., Jan. 31, 1903.

(*To be continued.*)

